# Economic Evaluation of Tobacco Treatments From the Screen ASSIST Lung Cancer Screening Trial

**DOI:** 10.1001/jamanetworkopen.2025.55332

**Published:** 2026-01-23

**Authors:** Douglas E. Levy, Shahzeb Hassan, Amy J. Wint, Caylin Marotta, Irina Gonzalez, Yuchiao Chang, Nancy A. Rigotti, Jennifer S. Haas, Elyse R. Park

**Affiliations:** 1 Mongan Institute Health Policy Research Center, Massachusetts General Hospital, Boston; 2 Tobacco Research and Treatment Center, Division of General Internal Medicine, Massachusetts General Hospital, Boston; 3 Harvard Medical School, Boston, Massachusetts; 4 Department of Dermatology, SUNY Downstate Health Sciences University, Brooklyn, New York; 5 Division of General Internal Medicine, Massachusetts General Hospital, Boston; 6 Health Promotion and Resiliency and Intervention Research Program, Massachusetts General Hospital, Boston; 7 Department of Psychiatry, Massachusetts General Hospital, Boston

## Abstract

**Importance:**

Integrating smoking cessation interventions within lung cancer screening (LCS) programs may significantly reduce smoking-related morbidity and mortality. Data on costs and cost-effectiveness of smoking cessation interventions in the context of LCS can foster the dissemination of effective treatments.

**Objective:**

To evaluate the cost-effectiveness from a health system perspective of 8 smoking cessation treatment strategies evaluated in the context of the Screen Aiding Screening Support In Stopping Tobacco (ASSIST) trial among patients undergoing LCS in 1 large health care system.

**Design, Setting, and Participants:**

The Screen ASSIST trial used a 3-way factorial design testing the effectiveness of 8 combinations of smoking cessation treatments at a health system in Massachusetts with 11 participating LCS sites. In this economic evaluation, costs and cost-effectiveness were evaluated prospectively and contextualized through sensitivity analyses. Participants included English-speaking and Spanish-speaking patients who smoked and were scheduled for LCS between April 2019 and July 2023.

**Interventions:**

Participants were randomized to 4 vs 8 counseling sessions, 2 vs 8 weeks of nicotine replacement therapy (NRT), and screening for social determinants of health (SDH) vs no screening.

**Main Outcomes and Measures:**

The primary effectiveness outcome was self-reported 7-day smoking abstinence at 6 months. Cost measures included both start-up and operating costs, with incremental cost per quit (ICQ) calculated relative to usual care.

**Results:**

Screen ASSIST had 642 participants (mean [SD] age, 64.0 [6.5] years; 358 female [55.8%]; mean [SD] 36.8 [19.4] pack-years; mean [SD] 16.2 [8.2] cigarettes per day). Start-up costs for the program were $131 371, primarily for electronic health record programming ($124 903). Operating costs for the intervention conditions ranged from $196 272 to $274 865 for 642 participants. The ICQ of 8 counseling sessions, 2 weeks NRT, and no SDH screening was $3050 (95% CI, $1286-4815), the most cost-effective condition. Per-patient costs and ICQ would be lower in health systems serving larger patient populations.

**Conclusions and Relevance:**

In this economic evaluation of smoking cessation treatment in LCS, 8-session counseling combined with 2 weeks of NRT was a cost-effective strategy, with a favorable ICQ relative to usual care and other treatment strategies evaluated. These findings are relevant for health systems considering integrating smoking cessation programs with LCS services.

## Key Points

**Question:**

Of the 8 smoking cessation strategies used in the Screen ASSIST trial among patients undergoing lung cancer screening, which was most cost-effective?

**Findings:**

This economic evaluation study including 642 participants found that 8 sessions of telehealth counseling combined with 2 weeks of nicotine replacement therapy was the most cost-effective intervention, with an incremental cost per quit of $3050. Sensitivity analyses illustrate potential cost differences in alternative settings.

**Meaning:**

These findings suggest that health systems can use this favorable incremental cost per quit and transparent cost estimation to promote wider implementation of the intervention.

## Teaser

This economic evaluation study examines the cost-effectiveness of 8 smoking cessation treatment strategies evaluated in the context of the Screen Aiding Screening Support In Stopping Tobacco (ASSIST) trial among patients undergoing lung cancer screening in 1 large health care system.

## Introduction

Tobacco smoking is responsible for 81% of lung cancer deaths.^1^ Since 2021, the US Preventive Services Task Force has recommended the use of low-dose computed tomography to screen for lung cancer in individuals aged 50 to 80 years with a 20–pack-year or longer history of smoking who are still smoking or who have quit smoking for fewer than 15 years.^2^ The guidelines recommend that anyone eligible for lung cancer screening (LCS) should also be offered smoking cessation services.^3^

The Screen Aiding Screening Support In Stopping Tobacco (ASSIST) trial was one of several smoking cessation trials conducted as part of the National Cancer Institute–funded Smoking Cessation at Lung Examination (SCALE) initiative. The study used a factorial design to both independently and jointly test 3 smoking cessation interventions in patients undergoing LCS.^4^ Under the factorial design, study participants were randomly assigned to 1 of 2 levels for each of 3 intervention components, resulting in 8 unique study conditions. The components were (1) offer of 4 LCS-tailored behavioral counseling sessions over 4 weeks or 8 sessions over 12 weeks, (2) offer of 2 or 8 weeks’ supply of nicotine replacement therapy (NRT) patches at no cost to the patient, and (3) offer or no offer of systematic screening for adverse social determinants of health (SDH) followed by referral to free community-based services, as needed. At the trial’s conclusion, statistically significant improvements were observed in the primary outcome (7-day self-reported abstinence at 6 months follow-up) for participants assigned 8 sessions of counseling vs those assigned 4 sessions.^5^ In addition, those assigned 8 sessions of counseling, 8 weeks of NRT, and SDH screening had significantly higher cessation rates than those assigned 4 sessions of counseling, 2 weeks of NRT, and no SDH screening (the most minimal treatment condition).

A preplanned economic evaluation was conducted for the Screen ASSIST trial. Given the multiple intervention components, the economic evaluation was designed to quantify the relative costs and benefits of increasing smoking cessation treatment intensity. This study focused on the cost of implementing and delivering the interventions as an important determinant of program adoption,^6,7^ estimating both the total costs and the incremental cost per quit (ICQ) of the various treatment scenarios from the perspective of a health system’s LCS program.

## Methods

Details of Screen ASSIST are reported elsewhere^4,5,8^; the study was approved by the Mass General Brigham institutional review board. Features of Screen ASSIST salient to this economic evaluation are presented here. This analysis followed the Consolidated Health Economic Evaluation Reporting Standards (CHEERS) reporting guideline.^9^ The Screen ASSIST trial tested the effectiveness of 3 separate intervention components on smoking cessation among patients scheduled for LCS who reported any cigarette use in the 30 days before enrollment. Eligible patients spoke English or Spanish, met Centers for Medicare & Medicaid Services coverage criteria for LCS, were not undergoing diagnostic evaluation for lung cancer, were able to give informed consent, were without significant psychiatric or cognitive impairment, had telephone access, and were willing and able to participate in study procedures. Patients did not need to be interested in quitting or taking NRT.

Recruitment occurred between April 2019 and July 2023 at 11 sites within the Mass General Brigham health care system in Massachusetts. Over that period, 12 025 patients were scheduled for LCS. With the assistance of an electronic health record (EHR) function created for this study, these patients were screened and invited by a research assistant to participate in the trial.^5^

### Interventions, Randomization, and Implementation

Study participants were randomized to 1 of 2 levels for each of 3 intervention components using a fully crossed factorial design.^10,11^ Counseling and NRT are well-established smoking cessation treatments.^11,12,13^ In addition, SDH screening and service referral may enhance the effectiveness of smoking cessation treatment by establishing a stronger rapport with patients and alleviating stressors that may inhibit cessation.^10,14^ Under this factorial design, there were 8 unique randomization conditions. The intervention components are discussed in the following subsections.

#### Telehealth Counseling

Smoking cessation counseling was delivered over videoconference or telephone by a certified tobacco counselor trained to use Motivational Interviewing, tailoring messaging to participants’ quit readiness and personal barriers to cessation. Participants were randomized to the offer of either weekly sessions over 4 weeks (4 sessions total) or weekly sessions over 4 weeks followed by 4 additional biweekly sessions over 8 weeks (8 sessions total). Initial sessions were designed to last 45 minutes (actual mean, 43 minutes), while follow-up sessions were designed to last 20 minutes (actual mean, 23 minutes). In practice, participants did not attend every session they were offered, and the duration of each session varied. The counselor met weekly with a psychologist supervisor to discuss program management and specific cases.

#### Free Nicotine Patches

Participants were randomized to receive an offer of either 2 or 8 weeks of NRT. Participants randomized to 2 weeks of NRT received all their medication at once. Those randomized to 8 weeks of NRT received a 4-week supply, then a second 4-week supply was encouraged by the counselor and fulfilled on request. Not all participants accepted the offer of NRT; of those assigned 2 weeks or 8 weeks NRT, 21% and 14%, respectively, declined. Sixty-one percent of those assigned 8 weeks NRT requested the second course. NRT was shipped via online retailer to participants’ homes.

#### SDH Screening 

Participants were randomized to the offer of screening for adverse SDH or not. Those accepting screening completed a web-based 16-question assessment at baseline from a service called FindHelp.^15^ The questionnaire assessed SDH domains, including housing, food, health care costs, employment, child and/or family care, legal services, loneliness, and social support, and identified free or low-cost services that participants could access locally. Assessment was self-administered or completed with the tobacco counselor, according to the participants’ preferences, and recommendations were sent to the participant. During follow-up sessions, the counselor offered screening again and checked on referrals accessed.

### Outcomes

For both the trial and the economic evaluation, the main effectiveness outcome was self-reported 7-day abstinence from smoking at the 6-month follow-up. As with some other SCALE studies, the trial was conducted during the pandemic, and biochemical confirmation was not possible.^16,17^ Intervention effects were calculated using an intent-to-treat approach.^5^

### Cost Measures

The cost of implementing the smoking cessation program was assessed for each of the 8 study conditions. The goal was to estimate costs that a future organization would incur when implementing and delivering programs as configured in each of the study conditions. These costs included start-up costs (eg, EHR programming or personnel training), the cost of enrollment, and the cost of delivering the intervention components.

The 2 main types of costs were personnel time and services or materials. Personnel time included initial expenses, such as time devoted to training and programming the EHR system to identify potentially eligible patients, as well as ongoing time devoted to screening, enrollment, and the delivery of intervention components. Most personnel time was valued on the basis of time spent and national median wages for each person according to their job type, drawn from the Bureau of Labor Statistics using 2021 data.^18^ Personnel costs included fringe benefits and overhead costs (eg, office space) (eTable 1 in Supplement 1). For implementation tasks completed by research assistants during the trial (eg, recruitment or mailing NRT), we assumed a real-world implementation would be performed by clinical staff receiving similar compensation. For the supervising psychologist role, we used the 90th percentile national wage for clinical psychologists to more accurately reflect compensation in this type of health care setting. Services (fees for counselor training or document translation) and materials (printed materials and NRT) were valued according to their research acquisition costs (eTables 2 and 3 in Supplement 1). We include document translation under the assumption that sites will customize their written materials and/or provide services to participants who do not speak English or Spanish. All costs, including in the Discussion, are updated to year 2025 US dollars.^19^

### Statistical Analysis

For each randomization condition, we tallied costs assuming all trial participants were assigned to that condition. Quantities of time and materials reflected what was used, not what was offered (eTables 4-6 in Supplement 1). For example, if a person was offered 8 counseling sessions but attended 6, only the cost of the 6 sessions used was included. We allowed for the possibility that use of one type of treatment (eg, NRT) might affect use of the other (eg, counseling), estimating costs for each randomization condition separately. To calculate the ICQ of any treatment strategy compared with another, we assessed the difference in per-person costs divided by the difference in quit rates. Because the lowest intensity treatment condition in the trial (4 weeks counseling, 2 weeks NRT, and no SDH screening) provided more services than might be offered under usual care, we also included a usual care condition whose cost was $0 and whose 6-month cessation rate was 2.7%, according to data for LCS-eligible smokers from the Health and Retirement Study (2018-2020; see eAppendix 1 in Supplement 1).^20^ ICQs were calculated using a league table approach.^21^ In instances where a condition was both less effective and more expensive than another option, it was determined to be dominated and, thus, an irrelevant option. Given the number of inputs with associated statistical uncertainty, SEs for cost estimates were obtained using Monte Carlo analysis (see eAppendix 2 and eTable 7 in Supplement 1 for details). ICQ 95% CIs were estimated using methods proposed by Fieller^22^ and Polsky et al.^23^

We assessed the sensitivity of our findings to several key inputs. First, we considered how the nominal cost per patient screened and the total costs would change over the course of 10 years if enrollment was reduced by half (eg, in a smaller health system) or doubled (eg, if the intervention was run as an external program serving multiple health systems). The base case annual enrollment value was the annual participant enrollment rate in the trial. These calculations make a conservative assumption that the health system needs to hire (and thus train) a new tobacco counselor every 5 years. Second, the incremental quit rate vs usual care in our base case was 18.3% (ie, 21.0% − 2.7%). However, the usual care quit rate was estimated from a different sample than the study population, so we also consider incremental quit rates as low as 10% and as high as 30%. Third, we considered costs to health systems that may already have EHR systems that can identify eligible patients (thus excluding information technology programming costs). Fourth, our calculations relied on the national median wages for each job type. We also reported ICQs setting wages at the 25th and 75th percentiles, nationally, to demonstrate variability across lower-wage and higher-wage markets. Monte Carlo analyses were conducted using Stata statistical software version 19.5 (StataCorp). All other calculations were conducted using Excel version 16.102.3 (Microsoft).

## Results

A total of 5707 patients confirmed current cigarette use, and 642 consented to participate in the trial (mean [SD] age, 64.0 [6.5] years; 358 female [55.8%]; mean [SD] 36.8 [19.4] pack-years, mean [SD] 16.2 [8.2] cigarettes per day). Costs to initiate the program, all upstream of intervention delivery, did not differ by randomization condition ($131 371). The largest was the cost of EHR programming to identify patients scheduled for LCS ($124 903). Costs of counselor training ($4476) and document translation to promote inclusion of Spanish-speaking patients ($1992) were smaller.

Table 1 displays the costs of the intervention conditions as implemented in the trial. Across the randomization conditions, the total operating costs for the study period ranged from $196 272 to $274 865 for 642 participants. Including the initial costs, total implementation costs ranged from $327 643 to $406 236, including EHR programming, and $202 740 to $281 333 excluding EHR programming. On a per-participant basis, operating costs ranged from $306 to $428. Enrollment averaged $164 per participant. Counseling ranged from $112 to $167 per participant and was generally more expensive than NRT ($23 to $91 per participant), while the cost of the SDH screening intervention was $6 per participant.

**Table 1.** Initial and Operating Costs to Implement and Deliver Screen Aiding Screening Support in Stopping Tobacco Interventions

Variable	Costs, $							
	4 Sessions				8 Sessions			
	2 wk NRT		8 wk NRT		2 wk NRT		8 wk NRT	
	SDH screening not offered	SDH screening offered	SDH screening not offered	SDH screening offered	SDH screening not offered	SDH screening offered	SDH screening not offered	SDH screening offered
Total costs (N = 642)	329 126	327 643	367 616	371 670	357 771	358 976	398 965	406 236
Initial costs	131 371	131 371	131 371	131 371	131 371	131 371	131 371	131 371
Counselor training	4476	4476	4476	4476	4476	4476	4476	4476
Electronic health record programming	124 903	124 903	124 903	124 903	124 903	124 903	124 903	124 903
Document translation	1992	1992	1992	1992	1992	1992	1992	1992
Operating costs (N = 642)	197 755	196 272	236 245	240 299	226 400	227 605	267 593	274 865
Operating costs per participant^a^	308	306	368	374	353	355	417	428
Enrollment	164	164	164	164	164	164	164	164
Treatment	144	142	204	210	188	190	253	264
Counseling	120	113	112	113	162	161	167	167
Medication	23	23	91	91	26	24	86	91
SDH screening	NA	6	NA	6	NA	6	NA	6

Abbreviations: NA, not applicable; NRT, nicotine replacement therapy; SDH, social determinants of health.

^a^ Because costs reflect services used rather than services offered, the cost of specific treatment components could vary across randomization conditions, reflecting possible interactions between services offered.

Table 2 shows the ICQ for each randomization condition compared with the usual care comparator using cessation rates for each group as reported in the trial (eFigure in Supplement 1).^5^ The condition with 8 counseling sessions, 2 weeks NRT, and no SDH screening had the lowest ICQ compared with usual care ($3050; 95% CI, $1286-4815). Because it also had the highest cessation rate, all other conditions were dominated, and sensitivity analyses focused on this intervention condition.

**Table 2.** Incremental Cost Per Quit by Randomization Condition Compared With Usual Care

Variable	Mean (95% CI)								
	Usual care	8 Sessions				4 Sessions			
		2 wk NRT		8 wk NRT		8 wk NRT		2 wk NRT	
		SDH screening not offered	SDH screening offered	SDH screening offered	SDH screening not offered	SDH screening not offered	SDH screening offered	SDH screening not offered	SDH screening offered
Incremental cost per participant, $	NA	557 (430 to 764)	559 (431 to 769)	633 (503 to 846)	621 (493 to 831)	573 (460 to 746)	579 (465 to 757)	513 (398 to 692)	510 (396 to 688)
Incremental cost per participant (excluding EHR programming), $	NA	363 (271 to 533)	365 (272 to 537)	438 (344 to 615)	427 (334 to 600)	378 301 to 515)	384 (305 to 526)	318 (239 to 460)	316 (237 to 456)
Quit rate, %	2.7 (2.0 to 3.4)	21.0 (12.7 to 31.5)	15.0 (8.0 to 24.7)	19.0 (11.0 to 29.4)	14.1 (7.3 to 23.8)	12.3 (6.1 to 21.5)	12.2 (6.0 to 21.3)	7.6 (2.8 to 15.8)	14.6 (7.8 to 24.2)
Incremental quit rate, %	NA	18.3 (9.4 to 27.1)	12.3 (2.3 to 16.9)	16.3 (7.6 to 24.9)	11.4 3.7 to 19.1)	9.6 (2.3 to 16.9)	9.5 2.4 to 16.6)	4.9 (−1.0 to 10.8)	11.9 (4.2 to 19.6)
Incremental cost per quit, $	NA	3050 (1286 to 4815)	4553 (1294 to 7812)	3890 (1539 to 6240)	5459 (1458 to 9460)	5948 (1184 to 10 712)	6109 (1260 to 10 959)	10 514 (−2638 to 23 665)	4283 (1240 to 7327)
Incremental cost per quit (excluding EHR programming), $	NA	1985 (764 to 3207)	2969 (752 to 5186)	2694 (1013 to 4374)	3750 (939 to 6561)	3927 (737 to 7117)	4056 (788 to 7324)	6524 (−1745 to 14 792)	2650 (691 to 4609)

Abbreviations: EHR, electronic health record; NA, not applicable; NRT, nicotine replacement therapy; SDH, social determinants of health.

**Figure 1.**
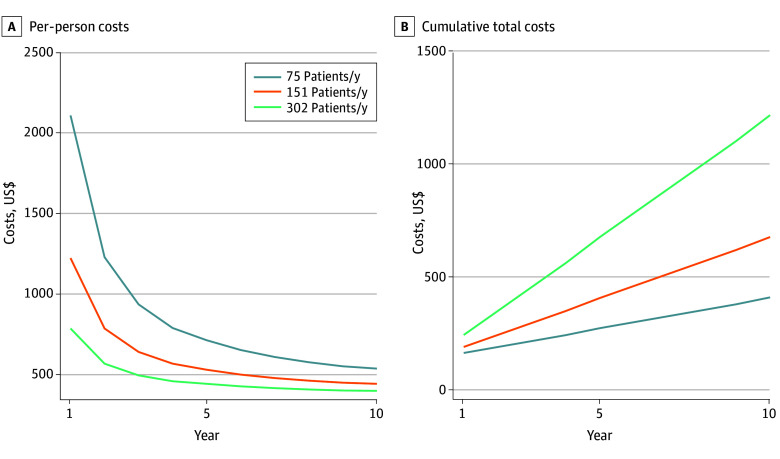
Costs According to Patients Enrolled Per Year Estimates were calculated for the condition with 8 counseling sessions, 2 weeks of nicotine replacement therapy, and no social determinants of health screening.

In sensitivity analyses, Figure 1 illustrates how costs were projected to evolve over the course of 10 years for different participant enrollment rates. The per-participant cost started high, reflecting how the initial costs were spread over a small number of patients initially. Over time, those costs were spread over increasing numbers of participants, and the per-participant cost leveled out. At the end of 10 years, the low-patient flow, base-case, and high-patient flow scenarios were projected to have total cumulative costs of $404 810 for intervention delivery to 750 participants, $672 822 for 1510 participants, and $1 214 273 for 3020 participants.

**Figure 2.**
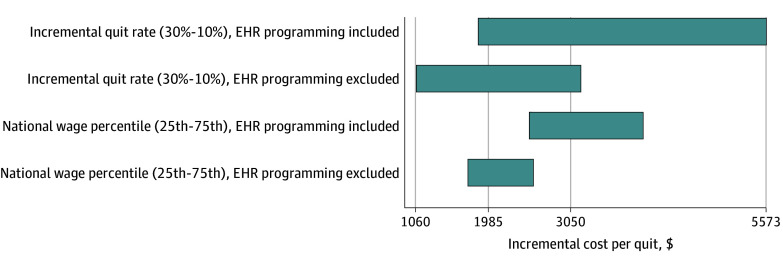
Sensitivity of Incremental Cost Per Quit to Alternate Incremental Quit Rate and Wage Assumptions Estimates were calculated for the condition with 8 counseling sessions, 2 weeks nicotine replacement therapy, and no social determinants of health screening. EHR indicates electronic health record.

Additional sensitivity analyses are presented in Figure 2. The ICQ varied with the incremental quit rate, which is uncertain due in part to the use of an outside sample to estimate the quit rate in usual care, and in part due to statistical uncertainty. Varying the incremental quit rate from 30% to 10% resulted in ICQs ranging from $1858 to $5573. In scenarios excluding additional EHR programming costs, the ICQ ranged from $1060 to $3181. Using the 25th and 75th percentiles of national wages to represent different labor markets, the ICQs ranged from $2550 ($1725 no EHR programming costs) to $3985 ($2570 no EHR programming costs).

## Discussion

The Screen ASSIST trial used a fully crossed factorial randomized trial design to determine the effects of 3 intervention components offered at minimal vs moderate levels, both individually and in combination, on smoking cessation outcomes for patients undergoing LCS. This economic evaluation found that only 1 of the treatment components (8 sessions of counseling delivered alongside provision of NRT patch) was more effective than its minimal counterpart.

Organizations interested in the cost of implementing the 8-session counseling intervention tested in Screen ASSIST should consider their local contexts. The single largest start-up cost for Screen ASSIST was programming the EHR to identify patients needing smoking cessation services. Over the 51-month study period, start-up costs constituted 38% of total costs. Such costs could be even higher if the program is implemented in a health system with an EHR that is challenging to customize, but might be substantially lower if the EHR functionality already existed or if it could be made easily portable across health systems. Payment models will also be relevant; our reported ICQ assumes the health system absorbs the cost of providing treatment, as it might under prospective payment (eg, an accountable care organization). Their costs might be lower under fee-for-service arrangements, but only if reimbursement is adequate. Wages also vary considerably across geographic areas, potentially increasing the ICQ by 31% or more in areas where wages tend to be at the high end of the distribution.

Few economic evaluations of smoking cessation interventions in the context of LCS have used empirical data on intervention costs and effectiveness. One such study was based on data from the Lung Screening, Tobacco, and Health (LSTH) trial,^24^ another member of the SCALE initiative. The LSTH study compared provision of 8 or 3 weeks of telephone-based smoking cessation counseling to no counseling at all among patients undergoing LCS, finding that providing no counseling at all was both more expensive and less effective than the counseling interventions. Focusing exclusively on intervention costs, the 3-week protocol cost $147 per participant and had a self-reported 9.4% quit rate.^25^ The 8-week protocol cost $391 per participant with a 10.3% quit rate. The ICQ of the 8-week vs 3-week protocols was $27 131. Although the per-participant costs were somewhat higher in Screen ASSIST, the intervention effectiveness was substantially higher, thus Screen ASSIST had a considerably more favorable ICQ.

Using a societal perspective, the incremental cost per quality-adjusted life-year (QALY) saved of the 8-week vs 3-week protocols based on biochemically verified cessation in LSTH was estimated to be $4893.^24^ Although cost per QALY saved was not estimated for Screen ASSIST, given that its ICQ was lower than that of LSTH, it is expected that the cost per QALY saved would be at least as favorable because the downstream costs and longevity benefits of cessation would be similar. Prior estimates of the value of smoking cessation in the context of LCS used simulation analyses and literature-based estimates of cessation effectiveness to estimate costs per QALY saved. We converted values from these studies to 2025 US dollars for comparability (see eAppendix 3 in Supplement 1), finding costs per QALY saved ranged from $778 to $49 797,^26,27,28^ with higher costs associated with higher intensity and more personalized counseling. All of these strategies are well within commonly accepted cost-effectiveness thresholds; the counseling provided in Screen ASSIST is likely to be among the more cost-effective options.^29^

### Limitations

Our analysis has certain limitations. First, effectiveness was assessed using self-reported rather than biochemically confirmed abstinence, potentially lowering ICQ values. Second, we assume that patients are offered smoking cessation counseling only as part of the index LCS event and not at subsequent routine or follow-up LCS tests. It is not clear from available data whether with repeated tobacco treatment offers, the ICQ would be higher (eg, if the most motivated individuals already quit smoking, yielding lower quit rates among those remaining) or lower (eg, most individuals require multiple attempts to quit smoking, so cessation rates would be higher on subsequent attempts). Third, the ICQ estimated in the current analysis was based on the experience of patients in a single health system. The costs and intervention effects may be different in health systems with different LCS care processes or patient characteristics. The sensitivity analyses presented in this study should buffer against such concerns.

## Conclusions

In an economic evaluation of the Screen ASSIST trial assessing smoking cessation interventions in the context of LCS, 8 sessions of telehealth cessation counseling, together with 2 weeks of NRT and no SDH screening, dominated other treatment combinations tested. At $3050, the ICQ of this intervention compared favorably to other smoking cessation interventions evaluated in the LCS setting and is likely to be highly cost-effective compared with commonly accepted thresholds for costs per QALY saved.

## Notes

Supplement 1. eTable 1. Wages plus fringe and overhead eTable 2. Training and EHR programming costs eTable 3. Other resources eTable 4. Patient identification activities conducted by research assistant eTable 5. Counseling delivery eTable 6. Medication fills eAppendix 1. Calculation of cessation rate for usual care eAppendix 2. Description of Monte Carlo analysis eTable 7. Stochastic parameters eFigure. Graphical presentation of incremental cost per quit eAppendix 3. Conversion of published cost values to 2025US$

Supplement 2. Data Sharing Statement
