# Supplementary material for: Economic Evaluation of Tobacco Treatments From the Screen ASSIST Lung Cancer Screening Trial
Source: JAMA Netw Open. 2026 Jan 23;9(1):e2555332. doi: 10.1001/jamanetworkopen.2025.55332 (PMC12831155; doi:10.1001/jamanetworkopen.2025.55332)
Supplement: Supplement 1. — eTable 1. Wages plus fringe and overhead eTable 2. Training and EHR programming costs eTable 3. Other resources eTable 4. Patient identification activities conducted by research assistant eTable 5. Counseling delivery eTable 6. Medication fills eAppendix 1. Calculation of cessation rate for usual care eAppendix 2. Description of Monte Carlo analysis eTable 7. Stochastic parameters eFigure. Graphical presentation of incremental cost per quit eAppendix 3. Conversion of published cost values to 2025US$ [file jamanetwopen-e2555332-s001.pdf]

Supplemental Online Content

Levy DE, Hassan S, Wint A, et al. Economic evaluation of tobacco treatments from the Screen ASSIST lung cancer screening trial. *JAMA Netw Open*. 2026;9(1):e2555332. doi:10.1001/jamanetworkopen.2025.55332

eTable 1. Wages plus fringe and overhead
eTable 2. Training and EHR programming costs
eTable 3. Other resources
eTable 4. Patient identification activities conducted by research assistant
eTable 5. Counseling delivery
eTable 6. Medication fills
eAppendix 1. Calculation of cessation rate for usual care
eAppendix 2. Description of Monte Carlo analysis
eTable 7. Stochastic parameters
eFigure. Graphical presentation of incremental cost per quit
eAppendix 3. Conversion of published cost values to 2025US$

This supplemental material has been provided by the authors to give readers additional information about their work.

© 2026 Levy DE et al. *JAMA Network Open*.

Model Inputs

eTable 1. Wages plus fringe and overhead

Bureau of Labor Statistics Occupation Code and Title	*Base case wage[a]*	*25th percentile[a]*	*75th percentile[a]*	*Study title, implementation role*	*Notes*
19-3033 Clinical and Counseling Psychologist	$120.53	$93.39[b]	$160.22[b]	Psychologist, training and supervising the counseling and administrative staff	Uses 90th percentile of the wage nationally, reflecting the training/expertise required for the role.
21-1023 Mental Health and Substance Abuse Social Worker	$45.44	$35.56	$62.91	Tobacco Treatment Counselor	
19-4061 Social Science Research Assistant	$45.81	$42.60	$58.29	Clinical Research Coordinator, recruitment	In practice, role might be fulfilled by a social worker and/or medical assistant.
	$98.89	$76.63[b]	$131.46[b]	IT Staff, programming EHR to identify patients scheduled for LCS who may be people who smoke	Based on local wages for this job type given the variability and imprecision in BLS titles in this category.

Abbreviations: IT, information technology; EHR, electronic health record; LCS, lung cancer screening; BLS, Bureau of Labor Statistics

[a] Based on May 2021 U.S. data obtained from the U.S. Bureau of Labor Statistics (www.bls.gov/oes/current/oes-nat.htm), except where noted. Added to this value is 32% fringe (based on local value) plus overhead, which includes the cost of office space. We assumed workers each occupied 64 square foot cubicles at a cost of $38.62/square foot in 2021$ (https://www.commercialedge.com/wp-content/uploads/sites/75/2021/12/CommercialEdge-Office-National-Report-December-2021-2.pdf). Overhead was prorated to an hourly rate. All ages are reported above in 2025$.

[b] Because the values used were not based on BLS median values, the 25th and 75th percentile values were estimated using the average relative distance of the 25th/75th percentiles from the median across all included job categories.

eTable 2. Training and EHR programming costs

Tasks	*Quantity/duration*	*Cost*
Initial		
Training courses	48 hours per counselor	$1791 tuition plus counselor time and ancillary (e.g., transportation) costs
EHR programming	1040 hours	IT staff time
Ongoing		
Counselor supervision	30 minutes per counselor per week	Counselor time, psychologist time

Abbreviations: IT, information technology; EHR, electronic health record

eTable 3. Other resources

Written patient materials (printing, postage)[c]	$14.08 per enrolled participant
Document translation	$1,592
Office space and related overhead	$3,002 per FTE per month ($17.32/hr)

Abbreviations: FTE, full-time equivalent employee

[c] Per enrolled participant costs include the costs of screening participants who did not enroll.

eTable 4. Patient identification activities conducted by research assistant

Research assistant's[d] pre-enrollment activities	*N*	*Time (hours)*
Screen list of patients scheduled for LCS	12,025	0.020
Chart review those who are identified as potentially eligible participants	5,707	0.132
Mail enrollment materials to potential eligible participants	5,707	0.020
Mail enrollment materials	642	0.130
Call potential participants, no answer	2,044	0.017
Call potential participants, left voicemail	4,241	0.033
Call potential participants, answered, not interested	3,645	0.083
Call potential participants, interested	753	0.208
Wrong number	21	0.017

Abbreviations: LCS, lung cancer screening

[d] In practice, role might be fulfilled by a social worker and/or medical assistant.

Though specific to the setting of this research-focused trial, recruitment costs and yields were included under the assumption that similar efforts would be required to engage individuals in such a program under non-research implementation conditions.

eTable 5. Counseling delivery

Counseling sessions (counseling and documentation)	*% Sessions completed*	*Average duration (hours)*
Session 1	94%	0.71
Session 2	84%	0.41
Session 3	74%	0.37
Session 4	74%	0.38
Session 5	73%	0.37
Session 6	63%	0.43
Session 7	62%	0.39
Session 8	60%	0.34

eTable 6. Medication fills

Packages[e] filled	Randomized to 2 weeks NRT	Randomized to 8 weeks NRT
0	68 (21%)	45 (14%)
1	251 (78%)	
2	3[d] (1%)	81 (25%)
4		188 (59%)
5		1[f] (0.3%)
6		5[f] (2%)

Abbreviations: NRT, nicotine replacement therapy

[e] Packages were 7mg, 14mg, or 21mg and contained 14 patches. Medications were ordered and shipped from an online vendor. Ordering time was negligible, and shipping costs were included in the price.

[f] In both arms there were a small number of instances where participants did not receive initial shipments and required replacements.

eAppendix 1. Calculation of cessation rate for usual care

The usual care quit rate was calculated using data from the 2018 and 2020 Health and Retirement Study (https://hrsdata.isr.umich.edu/data-products/public-survey-data). First, we identified people who currently smoked in 2018 and who met the eligibility criteria for lung cancer screening according to the 2021 United States Preventive Services Task Force guidelines (ages 50-80 with at least a 20 pack-year history of smoking). Current smoking was defined using question QC117. Pack-years was determined using number of cigarettes smoked per day (QC118) divided by 20, then multiplied by the number of years the person had been smoking (Q122). Cessation by 2020 (2-year quit rate) was determined using RC117; the proportion who were not smoking by 2020 was estimated accounting for the survey's complex design. We calculated the 6-month quit rate (the follow-up interval for Screen ASSIST) as follows, assuming that each of the four 6-month intervals of a 2-year period would have the same cessation rates:

$$QR_{6m} = 1-((1-QR_{2y})^{0.25})$$

Using the delta method, the standard error was computed as follows:

$$s.e._{6m} = 0.25(1-QR_{2y})^{-0.75}s.e._{2y}$$

eAppendix 2. Description of Monte Carlo analysis

In order to use Fieller's method for estimating a confidence interval around our ICQ estimates, it was necessary to estimate the variance of our estimated total costs. Given the large number of inputs affecting total costs, we used Monte Carlo methods to estimate the variance. Specifically, we re-estimated the total cost 100,000 times using inputs drawn from appropriate probability distributions for all stochastic parameters contributing to the total cost estimation. These are listed in eTable 7. For a number of parameters, we assumed that values might vary +/- 20%, and thus applied mutlipliers drawn from beta distributions. We assumed that wages across job types would vary to lower/higher values together. All statistical draws, including each multiplier, were made independently of one another. Using input values drawn from these statistical distributions, we re-estimated total cost 100,000 times. The variance of the total costs used in our ICQ confidence interval estimates was estimated as the variance of the 100,000 stochastically-derived total cost estimates.

© 2026 Levy DE et al. *JAMA Network Open*.

eTable 7. Stochastic parameters

Parameter	*Distribution*	*Source*
Number of outreach calls with no answer	Binomial (N=number eligible for program, p=2,044/5,707)	Study data
Number of calls, staff left a voicemail, no patient contact	Binomial (N=number eligible for program, p=4,241/5,707)	Study data
Number of calls, patient contacted, declined participation	Binomial (N=number eligible for program, p=3,645/5,707)	Study data
Number of calls wrong phone number, no patient contact	Binomial (N=number eligible for program, p=21/5,707)	Study data
Number of participants completing SDH form	Binomial (N=number eligible for program, p=74/323)	Study data
Number of participants sent SDH referrals by counselor	Binomial (N=number eligible for program, p=64/323)	Study data
Number of participants provided SDH resources to consult on their own	Binomial (N=number eligible for program, p=52/323)	Study data
Wages for counselor and research assistant	Empirical based on 10th, 25th, 50th, 75th, and 90th percentiles of national wage data	U.S. Bureau of Labor Statistics www.bls.gov/oes/current/oes-nat.htm
Amount of NRT provided	Empirical based on observed distribution in the study	Study data
Number of counseling sessions attended	Binomial (N=number eligible for program, p=arm-specific numbers of participants completing each session)	Study data
Multiplier[g] for random variation in psychologist and IT staff wages	Beta (alpha=2, beta=2, mean=1, range 0.8 to 1.2)	Assumed

[g] We used multipliers drawn from a beta distribution to add stochastic variability to certain simulation parameters. Each multiplier was drawn and applied independently. Wages for all jobs (including counselors and research assistants) were assumed to vary together (e.g., as they would in a particular job market).

© 2026 Levy DE et al. *JAMA Network Open*.

Additional detailed results

eFigure. Graphical presentation of incremental cost per quit

Illustrates that all but 8C, 2w, SDH(-) are dominated.

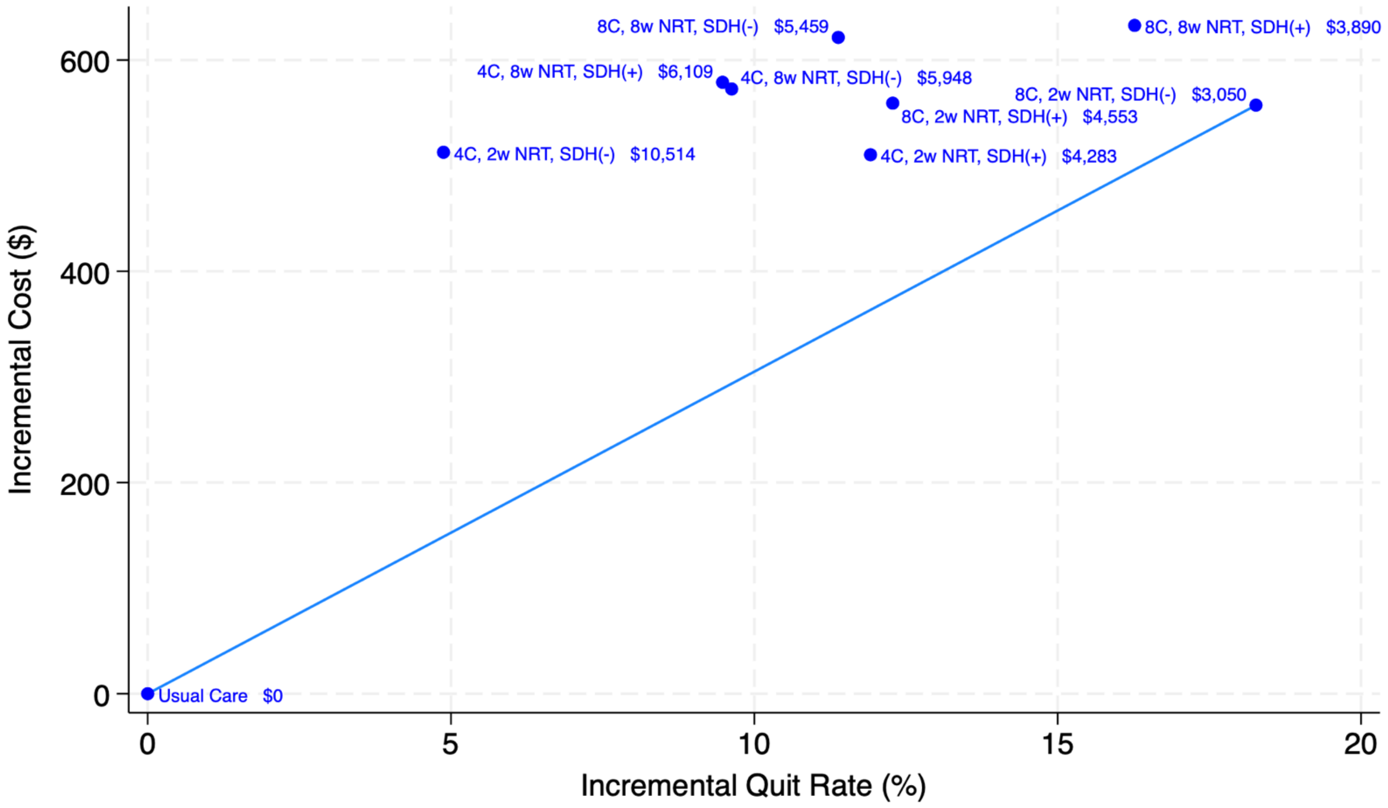

Abbreviations: 4C = 4 counseling sessions, 8C = 8 counseling sessions, 2w NRT = 2 weeks nicotine replacement therapy, 8w NRT = 8 weeks nicotine replacement therapy, SDH(-) = social determinants of health screening not offered, SDH(+) = social determinants of health screening offered

© 2026 Levy DE et al. *JAMA Network Open*.

eAppendix 3. Conversion of published cost values to 2025US$

For values published in US dollars, amounts were updated using the All Urban Consumers (CPI-U) consumer price index (https://www.bls.gov/data/inflation_calculator.htm) from January of the source year to January of 2025. For values published in Canadian dollars we used a method suggested by Turner et al.[h] Amounts were first adjusted for inflation to 2025 using a Canadian inflation calculator (www.inflationcalculator.ca), then converted to US dollars using the exchange rate for that year (https://www.bankofcanada.ca/rates/exchange/annual-average-exchange-rates/).

[h] Turner HC, Lauer JA, Tran BX, Teerawattananon Y, Jit M. Adjusting for Inflation and Currency Changes Within Health Economic Studies. Value Health. 2019 Sep;22(9):1026-1032. doi: 10.1016/j.jval.2019.03.021. Epub 2019 Jun 14. PMID: 31511179.

© 2026 Levy DE et al. *JAMA Network Open*.
