# Supplementary material for: Economic Evaluation of Tobacco Treatments From the Screen ASSIST Lung Cancer Screening Trial
Source: JAMA Netw Open. 2026 Jan 23;9(1):e2555332. doi: 10.1001/jamanetworkopen.2025.55332 (PMC12831155; doi:10.1001/jamanetworkopen.2025.55332)
Supplement: Supplement 2. — Data Sharing Statement [file jamanetwopen-e2555332-s002.pdf]

Data Sharing Statement

Levy. Economic Evaluation of Tobacco Treatments From the Screen ASSIST Lung Cancer Screening Trial. *JAMA Netw Open*. Published January 23, 2026.
doi:10.1001/jamanetworkopen.2025.55332

Data
Data available: Yes
Data types: Deidentified participant data, Data (not involving human participants)
How to access data: Participant data from the Screen ASSIST trial is available in the Harvard Dataverse (see doi:10.1001/jamainternmed.2024.8399). Data (not involving human subjects) consists of input data for cost estimation which is included in the Supplement with this manuscript submission.
When available: With publication

Supporting Documents
Document types: None

Additional Information
Who can access the data: Researchers whose proposed use of data has been approved.
Types of analyses: For any purpose
Mechanisms of data availability: With investigator support
